# An anthropological analysis of the perspectives of Somali women in the West and their obstetric care providers on caesarean birth

**DOI:** 10.3109/0167482X.2010.547966

**Published:** 2011-02-04

**Authors:** Birgitta Essén, Pauline Binder, Sara Johnsdotter

**Affiliations:** 1 Women's and Children's Health, International Maternal and Child Health (IMCH), Uppsala University, Uppsala, Sweden; 2 Faculty of Health and Society, Malmö University, Malmö, Sweden

**Keywords:** Caesarean refusal, emergency caesarean, pregnancy strategies, immigrant, maternal care guidelines

## Abstract

We explored the perceptions of 39 Somali women and 62 obstetric care providers in London in relation to caesarean birth, as borne out of a paradox we recognised from evidence-based information about the Somali group. Socio-cultural factors potentially leading to adverse obstetric outcome were identified using in-depth and focus group interviews with semi-structured, open-ended questions. A cultural anthropology model, the *emic/etic* model, was used for analysis. Somali women expressed fear and anxiety throughout the pregnancy and identified strategies to avoid caesarean section (CS). There was widespread, yet anecdotal, awareness among obstetric care providers about negative Somali attitudes. Caesarean avoidance and refusal were expressed as being highly stressful among providers, but also as being the responsibility of the women and families. For women, avoiding or refusing caesarean was based on a rational choice to avoid death and coping with adverse outcome relied on fatalistic attitudes. Motivation for the development of preventive actions among both groups was not described, which lends weight to the vast distinction and lack of correspondence in identified perspectives between Somali women and UK obstetric providers. Early booking and identification of women likely to avoid caesarean is proposed, as is the development of preventive strategies to address CS avoidance.

## Introduction

Somali women of reproductive age have been steadily migrating into the West since the beginning of the 1990s, due to civil war and political unrest in their homeland. The estimated figures for total Somali resettlement suggest that nearly 240,000 Somali people currently reside in western countries [1]. A number of investigations have been conducted, which explore the obstetric care experiences of Somali women within a specific western context, as well as their maternal outcomes in general. Such findings are important for supporting the migration experience of these women, but they also lend evidence-based knowledge to the overall goal of improving maternal health as outlined in the UN Millennium Development Goal 5 [2]. Indeed, it has been recently postulated that enough information is now known to put global knowledge into action [3].

Qualitative studies have attempted to learn what Somali women want from their westernmaternal care. Arising from this global effort is a consistent finding: many of the Somali women who have been interviewed hold very negative attitudes about caesarean delivery. In Sweden, such attitudes are, for example, said to result from anxiety about dying or about complications to future pregnancies that might arise from the operation [4]. The topic of caesarean birth has also been described by Somali women living in the USA in association with fear and apprehension [5]. Women living in Norway expressed not only a fear of the procedure, but also a general dissatisfaction and scepticism with what they saw as an unwarranted operation that was also being performed too frequently [6]. In another US study, Somali women were shown to prefer maternal care from obstetricians who are ‘conservative’ regarding the decision to perform this procedure [7], while a Canadian study showed that despite not being wanted or chosen as a mode of birth, caesarean section (CS) was performed often for this participant group [8]. Epidemiological studies, on the other hand, indicate that rates of CS are elevated for Somali women across western settings after adjusting for various background variables and are well above figures for country-born mothers [9–12]. One of these studies further identified emergency CS and showed a 3-fold higher risk for Somali women compared to ethnic Norwegian women, after adjusting for maternal age and parity [12].

Concurrently, Somali women who are now living in high-resource countries show an increase in adverse obstetric outcomes for perinatal mortality [11–13]. Somali women are also presumably represented within reports of elevated maternal mortality among immigrant African women in Europe. In the UK, for example, over the period 2003–2005, immigrant African women were identified in the national Confidential Enquiry into Maternal and Child Health (CEMACH) Report as having a maternal mortality rate nearly six times higher than White women [14]. This figure is double the number of deaths from the previous CEMACH Report for 2000–2002. Increased maternal mortality rates for African immigrants have also been reported in the Netherlands and France [15,16], and risk for severe acute maternal morbidities has been shown as elevated among sub-Saharan African women living in the Netherlands relative to rates shown for native-born women [17].

A review of this vast base of evidence suggests a paradox: women who hold negative, fear-based attitudes towards CS simultaneously show an elevated frequency of the procedure (relative to country-born women) as well as a potential for heightened risk of maternal and/or perinatal complications and mortality. The reasons for elevated rates of CS among African immigrant women over those reported for country-born mothers are not well understood within the specialty of obstetrics and gynaecology. Furthermore, for Somali women in particular, it seems highly relevant to characterise why some of these women are strongly apprehensive and fearful towards CS, despite presence of vaginal bleeding or other signs of acute medical indication. We contend that, as yet, not enough empirical information is available, which appreciates the crucial link between Somali women's attitudes towards the CS procedure, the western obstetric care they receive and the potential for negative outcome. This study therefore aims to address the relationship between Somali women and their western obstetric care providers. The attitudes, perceptions, beliefs and experiences of both groups will be explored in relation to CS, with an additional aim to identify potential factors which might lead to adverse obstetric outcome.

## Methods

### Study procedures

Ethical approval for undertaking this study was approved by the Riverside Research Ethics Committee in London, 06/Q0401/15. In-depth individual and focus group interviews using semi-structured, open-ended questions were performed by an obstetrician (BE) and an anthropologist (SJ). Each interview was tape recorded and took approximately 30–90 minutes. All interviews were performed following individual written informed consent. At the beginning of each interview, the researcher conducting the interview explained that participation could be declined at any time and without explanation.

Participants were sought throughout Greater London between 2005 and 2006. These included immigrant Somali women, who had had at least one child within the British health care system and who were living within the study area at the time of data collection, and maternal care providers with professional affiliation as a doctor or midwife at five hospitals within the study area. Providers also had extensive experience in caring for women of British and non-British ethnic backgrounds. Thirty-nine Somali women participated in 23 individual interviews and four focus group interviews, comprised of 2–6 participants. A female Somali interpreter was used during 10 individual interviews and three focus group interviews to directly translate Somali into English. The age range of the women was 18–48 years, and time spent in the UK ranged between > 1 year and < 20 years. Parity among the group was 1 to 10 children. Of the total women interviewed, 14 described having what we interpreted as emergency CS for fetal indication for at least one birth, whereas eight women described either a non-emergency but acute CS or a planned CS due to maternal indication. One woman elected for CS early in her pregnancy for at least one birth. One woman experienced both emergency CS and planned CS for two respective births. The 62 participating maternal care providers represented multiple ethnic profiles (4 Somali, 34 other African or Caribbean, 21 White British and 3 Asian). We defined the ethnic profile of providers as country of birth. Fifty-two individual interviews and three focus group interviews (2–5 participants each) were conducted among the care providers.

### Recruitment

The snowball sampling technique [18] was used to recruit some of the Somali participants at the community level, and saturation of referrals was reached at 36 women. The snowball referral was initially arranged by 10 female Somali ‘culture brokers’, who were later commissioned as advocates and interpreters. Culture brokers are persons well known within a community or who are familiar with the culture and habits of the study population [19]. These women acted on behalf of the researchers to set up the first contacts and focus groups in the homes of Somali women throughout the study area and assisted the researchers to follow-up on individual interviews around different neighbourhoods. All Somali women who were recruited in this manner were given the choice, by a culture broker or by the head researchers upon explanation of the study design, to be interviewed individually or to take part in a focus group. The choice was left to the individual women due to the private nature of the topic. The three remaining Somali women were recruited purposively [20] at the hospital level via the head midwife or by an on-call obstetrician for individual interviews. In these few cases, the researchers, who were not members of the hospital care staff, were notified and received permission to approach each woman while at the maternity ward. Without regular maternal care staff in the room, the researchers introduced the study and asked if the women wished to be interviewed. Additionally, the participating care providers also comprise a purposive sample. The providers at the various study hospitals were introduced to the project by a posted sign at the maternity ward, which invited all interested providers to attend an information meeting given by the research team. At this occasion, interested providers were invited to interview, and a date was made to take place at a location within the hospital. The providers were given personal choice to be interviewed individually or by focus group, as based on availablity.

### Interview themes

Questions posed to Somali women focused on themes such as general health care experience within the British system, value judgements or notions of belief around medical care procedures and routines, their own pregnancy and post-pregnancy care strategies, perceptions of antenatal care and interaction with providers. Questions posed to providers focused on the provision of care to immigrant and non-immigrant women and aimed to identify perceptions about women's patterns of care-seeking behaviour, as well as to describe care experiences with women during the antenatal or labour period; and identify perceptions and experiences regarding the management of care of immigrant women.

### Theoretical perspective

Borrowing from cultural anthropology, the terms emic and etic are commonly applied to different kinds of knowledge or understanding, especially when cultural gaps exist. In the present context, an *emic* perspective refers to meaningful interpretation from both patient and provider as developed within his/her own culture, while an *etic* perspective refers to the recipient observer who, in objective assessment as the research team, tries to remain culturally neutral [21].

### Data analysis

This study relied on a framework of naturalistic inquiry [22]. Analysis began during the early interview phase in order to develop additional open-ended questions, which were then incorporated into subsequent interviews. For final analysis, all tape-recorded interviews were transcribed into text. A second anthropologist (PB) analysed the written transcripts, where the overall similarities, patterns and differences across respondents were identified and interpreted into intuitive categories, and then analysed further for interpretation relative to caesarean delivery so as to glean a picture of individual lived experience. The resulting intuitions were defined as perspectives that support the theoretical underpinnings of the *emic/etic* model. This design is intended to avoid separating the study method from the conceptual theory supporting the research themes [23].

## Results

**Figure 1:**
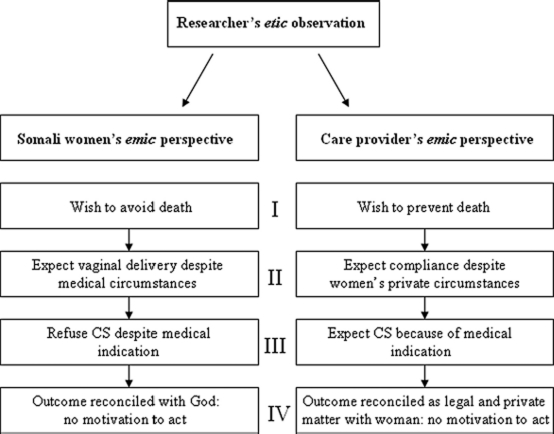
The *emic* perspectives of Somali women and their obstetric providers, as identified from the *etic* position of the researchers, show potential for conceptual misunderstanding in maternal care in relation to CS.

With respect to CS, Somali women's *emic* definition of required care and treatment do not correspond to the provider's *emic* biomedical expectations. Figure 1 summarises the opposing attitudes, beliefs and perceptions about prior knowledge of both women and providers and highlights key areas that are discordant and potentially likely to inhibit open interaction between the groups.

### Avoiding death vs. preventing death

The Somali women in our study believed that CS delivery might likely result in maternal death, while the providers identified CS as preventive care that is intended for saving the life of mother and infant. Nearly all women who discussed CS directly, or who discussed it tangentially in relation to someone they knew, consistently expressed fear or apprehension about the procedure. ‘You know, you just don't know where you'll be: the life or the death. That's what makes me so scared (Somali woman 16, three children).’ Most related it to knowledge, either personal or through hearsay, of someone who had not survived the procedure in Somalia. ‘In Somalia … women die all the time. I was really very worried during my pregnancy … if you are pregnant in Somalia you are on the ‘curse’ between life and death. You don't know what is going to happen to you. That is what the old, like my grandmother, say. These are common words in Somalia (Somali woman 5, three children).’ Negative attitudes also took the form of denying the care provider's assessment, and some women made the decision not to return to the same clinic for future care while others simply chose not to follow the provider's advice. On the other hand, while perceptions expressed by women emphasised avoidance under most circumstances, some of the women who had experienced CS had, in retrospect, a reserved openness for the procedure in relation to their relief at having had a healthy baby. In these women, however, recall of the birth event included a retrospective belief that their surgery was unnecessary or that they would have preferred to avoid exposure to analgesia. Complaints were made about having to manage recovery time against family life, and such women also had rather clear complaints about their postoperative care. ‘Everything was okay except for the service at the hospital … it was very, very bad. I've given birth to two babies at this hospital [but] when I gave birth by Caesarean, no one even watched over the baby. When I asked for help to put on my shoes, [the nurse replied] “Today I will help you, but on another day, don't ask me … take by yourself!” … I cried … (Somali woman 9, eight children).’

Most providers identified awareness of the negative attitudes held by Somali women in relation to caesarean delivery, and they based this on personal encounters with Somali patients or from knowing colleagues who had presented cases during clinical review. ‘I've met many Somali women and men who are very afraid of Caesarean section … (White British doctor).’ All Somali providers considered women's apprehension about CS to result from a fear of dying: ‘Anybody, very few people who had an operation actually survived. So it was a bad thing, to be avoided at all costs … When we came to the UK we brought this idea with us (Somali doctor).’ The non-Somali providers who had direct experience with the issue of CS among this population of women described situations of stress for health personnel attending the case. ‘Somalis don't like Caesarean sections even in direct emergencies. That's a very difficult situation because it is a very demanding emergency where the delivery has to occur within a few minutes, and if there is a lot of resistance on the part of either the patient or the relatives it puts the team who is managing under a lot of pressure … It is still in my memory and it is really traumatising … (Asian doctor).’ Stress as well as frustration was expressed by some providers as a generalised negative attitude towards the patient group. ‘The most problematic group among immigrants … because [Somalis] do not understand the high risks for problems and they don't understand the message we are giving to them or our preventive way of thinking regarding maternal health care (White British doctor).’ A number of providers commented on an overall discordant experience with Somali women. ‘Somali women are not into preventive medicine at all (White British doctor).’

### Ensuring vaginal delivery vs. expectations of compliance

At onset of labour, a number of women described an intention to postpone going to hospital. ‘If the baby turns then I will be able to give birth naturally, but if it doesn't they will have to operate … I waited until I had the contractions and was open 5 centimetres. Then I went to hospital (Somali woman 10–2, six children).’ Many women reported that recommendations about waiting to go to hospital came from other women in the Somali community. ‘Here in London, when you have long suffering … the baby is taken by Caesarean. Therefore, I am afraid that if I arrive early and take a little time … I will have to be operated … It is better to wait … Those who have given birth here have told me (Somali woman 19, four children).’ Some hearsay information among the Somali women was specific towards avoiding CS. ‘I have given advice: Do not go early to the hospital. You have to wait until the last minute. This is the rule in Britain that it becomes twice Caesarean (Somali woman 25, three children).’ A few providers were aware of the Somali pregnancy strategy to avoid hospital if there was a risk for CS. ‘There was a Somali patient who was booked first for a section and she asked them if she could go home to collect her stuff and she never came back … I went to see her at home and she said “I am going to have a vaginal delivery. I am not having Caesarean. I know I have lost two babies, but never mind, this one is going to be normal.” [Eventually she waited to go to hospital until she was dilated to] 9 centimetres … and she had a vaginal delivery. She came out and said, “I told you …” (Other African midwife).’

### Refusal of CS vs. medical indication

‘I refused and just kept pushing and pushing … All the Somalis, at least the ones I know, hate the Caesarean … I didn't want Caesarean (Somali woman 5, three children).’ Some providers explained that because consent for CS is required, they must oblige whatever decision is made even if it means loss of life. Gaining consent for CS was also described as having unexpected obstacles. ‘Sometimes you may feel that you can quite easily convince the mother, but her husband is the barrier (Other African doctor).’ Complications were described as not knowing the point of problem onset and thus not being able to assess the severity of fetal compromise. Unnecessary barriers were also targeted in their descriptions. ‘We could have easily prevented this three hour delay … the woman arrived late and then she was not able to understand what we were talking about, and by the time we called for an interpreter it was too late (Asian doctor).’

Providers who were already aware of Somali pregnancy strategies in relation to avoiding CS described experiences when the mother outright rejected care after being advised to undergo CS. ‘It doesn't matter what we tell them, whatever the consequences are, it's the work of Allah. We don't have any influence at all (Other African midwife).’

### Reconciliation vs. motivation to act

If the birth outcome ended in tragedy, the coping perspective of the woman and her family was described as relying on religious belief, while the providers' coping perspective was expressed towards the family, as a private matter. ‘There is a lot of mixed guidance that comes out from the obstetricians and gynaecologists and you know there is a lot that your legal department gets involved in … but at the end of the day, I can only answer the patient's questions about whether she needs a section and if so, why … I can't make her have a section and neither can the doctor … it is the woman's decision (White British midwife).’ The notion of coping with the death of an infant, while considered absolutely tragic and undesirable, was also considered to be out of the women's hands, and a number of women expressed contentment at having their religious beliefs to rely upon. Conversely, the providers rarely expressed any form of understanding over the loss of the child, but still tried to come to terms with the situation. ‘I don't think we have any guidelines here for how to manage someone who doesn't agree with you (White British doctor).’ Nevertheless, all providers who had been involved in CS refusal commented that they had informally discussed their experience among their colleagues; however, no providers could remember any resulting guidelines on how to prevent such situations. ‘I don't know if something is brought up in meetings or whether there are guidelines on how to handle different expectations … Refusal itself doesn't get talked about. Providers are disappointed and so on, but they just go ahead in the end (Caribbean midwife).’

## Discussion

The majority of Somali women in our study supported a strong association between caesarean birth and maternal death [4,24]. For these women, attitudes supporting fear and apprehension seemed to be situated in a rational fear of dying. Such fear is rational to individuals when appreciated against a pre-migration background, where African women have the highest risk for maternal death, and high mortality rates are compounded by high pregnancy rates [25]. In general, the maternal care providers were aware of Somali women's fears, but they did not seem to consider this perspective within a post-migration context. Findings also support that the care providers viewed adverse outcome as the result of a decision made by the woman (which is based on the legal aspects surrounding consent) and coping with the matter as a private family issue. On the other hand, the women coped by having a fatalistic attitude that relied on their own personal belief system. These distinct coping strategies strongly suggest that motivation for the development of preventive action is missing in relation to the severity of the obstetric outcome. The women deal quietly with the circumstance, while the care providers might consider the weight of importance differently than if a morbid or mortal result had been steered alone by medical indication. Overall, Somali women's perspectives of required care and treatment in relation to CS do not correspond to maternal care provider's medical expectations and vice versa.

Approaching the dataset from an *etic* perspective has made clear a vast distinction between the *emic* perspectives of both the Somali women and the London maternal care providers. Using the *emic/etic* model to explore Somali pregnancy experience as well as the providers' care giving experiences has allowed us to identify perceptions about caesarean birth that are, from an individual informant's point of view, meaningful and valid. By doing so, contrasts and contradictions are thus more easily defined between the two groups [26]. A potential limitation is generalisability, and we therefore validate a number of our findings based on coherence with other qualitative studies conducted globally on this immigrant population [27]. Further, Somali women tend to support a strong oral culture [7,28], and shared knowledge might have a tendency to spread quickly and influence many women within the same group, which has potential to bias the snowball sample. Consideration was therefore given during the analysis to ensure that information relayed about experiences in the UK was understood from the individual's point of view [23], while second-hand or descriptive information about others' UK experiences was kept distinct.

Mistrust among Somali women in the western maternal care setting has been identified in numerous studies [5,28,29]. In this context, the women seemed to feel strongly about being told what to do around the topic of CS, and they held negative impressions about the system that practices it. Most women also held a general attitude in favour of having their own voices heard during the overall pregnancy care, which is a finding consistent with the literature [29]. Several of our participants also stated that no intervention was necessary as long as things appeared to be going well with the pregnancy and that a woman knows best how to manage her own pregnancy. Our maternal care providers, on the other hand, showed that they are likely to view the latter attitudes as being associated with higher risk for obstetric complications and adverse outcome. The gap presented here between these two perspectives causes us to question how providers would convey to their Somali patients about the need for seeking help if something in the pregnancy seemed questionable or problematic or about being open to receiving appropriate obstetric interventions. One audit conducted among East African immigrants living in Sweden found a strong association between adverse perinatal outcome and maternal pregnancy strategies that either did not incorporate accurate risk-assessment or did not allow for appropriate obstetric interventions [30]. This study also concluded that ‘refused Caesarean section despite medical indications’ was the most common cause of avoidable perinatal death. Taken together, we ultimately conclude that there exists a strong potential for unanticipated complications in this western care setting, which provides a platform for further investigation into the factors behind them. Moreover, the paucity of advice available to practitioners might be due to the likelihood that qualitative research in this area is in need of much attention. How should the maternal care provider respond to this special situation when a woman has a valid perspective of apprehension based on fear and refuses the treatment, which is, conversely, the treatment most likely to prevent the adverse outcome she fears?

To our knowledge, CS avoidance, as resulting from misconceptions within the patient-provider interaction, has not been previously reported in studies examining adverse pregnancy outcome among Somali immigrants. However, Chalmers and Omer-Hashi [31] discussed the high incidence of unnecessary CS among women who showed no apparent indication for CS, but who had undergone female genital mutilation (FGM). Whereas, their care provider guidelines caution that women with FGM are potentially unlikely to return to the hospital for antenatal care if advised about a need to have CS (due to fear or an association of the procedure with death), these authors also question whether Canadian caregivers believe that CS is a medically necessary procedure for women with FGM. Of interest to our findings, neither the women nor the care providers described FGM as a concern for their maternal care experience. We interpret this absence as a positive sign that knowledge about FGM has been effectively incorporated into the UK health service. FGM education for maternal care providers has been fully active in the UK since 1983, and antenatal guidelines are regularly updated and include advice for providers to identify FGM early by means of sensitive enquiry, and to follow-up with an intrapartum care plan [32]. Further, within the Greater London area, several specialised clinics provide FGM-related services and education to African immigrant women [33].

As a point of departure from standard maternal care, such well-established clinics – as well as similar clinics found internationally – might provide a proper locale for Somali women by making routine the topic of CS delivery during antenatal consultations. This becomes especially important when the situation is not life threatening. The number of Somali women in our study who had had a discussion with a care provider about CS before the birth was very low. Chalmers and Omer-Hashi [8] reported that nearly 25% of the CS cases in their study had had no discussion of procedures or options before the actual moment of birth. This same study also found that CS was wanted by less than 1% of the participants but was experienced by over 50% of the 432 women they interviewed. In the time since their reporting, our discussion indicates that not much has changed. Ultimately, the findings support implementation of specific needs consultation for Somali women, where extra care can initially be taken to present and discuss information about common and routine interventions, such as ultrasound scan and fetal monitoring. The providers should be sensitive to the idea that introducing such interventions into pregnancy care has the potential to leave a Somali woman with a perception that something is wrong with her pregnancy [4]. Further, indications from our findings and the qualitative literature at large suggest a possibility that Somali women will consider as justifiable a switch of their obstetric care provider during the antenatal period. Therefore, CS, induction of labour and methods of pain relief during delivery should be presented and discussed with women and their partners as early as possible during antenatal consultation – at a time when the information is not personalised. Because of high potential for misconception between Somali women and care providers on the topic of caesarean delivery – and interventions in general – as well as concerns of Somali women about their language abilities during maternal care [34], the use of professional interpreters is likely required. However, optimised use of interpreters for the care of Somali women during pregnancy and labour has yet to be sufficiently defined across the literature.

**Figure 2:**
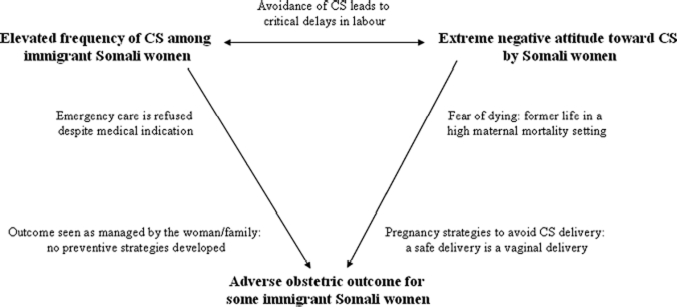
The CS paradox. Caesareans are being performed too often and too late in Somali mothers who are known to be negative towards the procedure and who show high risk for adverse obstetric outcome.

We have presented here several factors, which might help to explain how a procedure that normally produces optimal outcomes can result in adverse conditions among this immigrant group. The elevated rate of caesarean in paradoxical relation to open refusal of the procedure requires early identification of potentially high-risk cases, given especially the increased likelihood for complications and mortality (Figure 2).

The women's fear of dying remained a significant point of discussion, despite now living in a high-resource setting and irrespective of the time spent out of Somalia. Future studies on this topic could include more precise consideration of the length of time a woman has lived in the western context in relation to such persistent attitudes. Furthermore, a large number of our informants also described participation in the local Somali social network with regard to their pregnancy care and indicated that they perceived as important the hearsay information circulating among women about CS and the caesarean experience. This finding lends weight to the probability that Somali women prefer and are more likely to show willingness for pregnancy care as based on verbal communication [28,29]. Reliance upon an active social network also addresses decision-making for Somali women, and suggests that with regard to caesarean delivery, the final decision may likely go beyond the women. Notwithstanding, our care providers described frustration and an inability to convince women for emergency CS due to significant input from the partner and extended family members. Our understanding about Somali women's decision-making during obstetric care would thus benefit by an exploration of how critical decisions are influenced by important social contacts. To date, the western medical community has yet to completely understand CS avoidance among Somali women, despite presence of the phenomenon for over two decades. Preventive strategies remain limited on the essential topics of caesarean avoidance and refusal. This study, conducted in the UK, shows that Somali women as well as their maternal care providers remain highly vulnerable for experiencing emergency CS in the high-resource, western setting.

## Current knowledge on this subject

We reviewed the available literature and observed a paradox:Somali women living in the West are shown to hold very negative attitudes towards caesarean birth. After adjusting for various background variables, there is an elevated tendency for caesarean section among Somali women relative to western, country-born women. African women living in the West, and Somali women in particular, have been shown to have an increased potential for adverse obstetric outcome.

## What this study adds

Somali women make what are perceived to be rational choices to avoid caesarean birth. Preventive strategies among western obstetric care providers remain limited on the essential topics of caesarean avoidance and refusal. Somali women living in the West, as well as their obstetric care providers, remain highly vulnerable to experiencing potentially avoidable emergency CS despite the high-resource, western setting.
